# Are Attributes of Pregnancy and the Delivery Room Experience Related to Development of Autism? A Review of the Perinatal and Labor Risk Factors and Autism

**DOI:** 10.1155/2014/290837

**Published:** 2014-10-01

**Authors:** Naveen Dhawan, Blaze Emerson, Romana Popara, Catherine Lin, Adam Rawji, Rita Zeiden, Leeda Rashid, Pwint Phyu, Jaya Bahl, Vineet Gupta

**Affiliations:** ^1^Nova Southeastern University, Health Sciences Division, Fort Lauderdale, FL 33314, USA; ^2^CEP America, Emeryville, CA 94608, USA; ^3^Department of Medicine, University of California San Diego (UCSD), 200 West Arbor Drive, MC 8485, San Diego, CA 92103, USA

## Abstract

Autism is a neurodevelopmental disorder marked by severe deficits in social communication and interactions. It is a complex condition that lacks an established preventive method, warranting a need for research to identify possible environmental triggers. The identification of external factors particularly perinatal risk factors forms the initial critical step in preventing and alleviating risks. We conducted a literature review to assess evidence suggested in the worldwide literature. Perinatal risk factors that have a suggested association include *β*2 adrenergic receptor agonists, labor induction and augmentation, maternal infection and disease (i.e., antiphospholipid syndrome), antiepileptic drugs, cocaine use, and oral supplements. Smoking has not been found to have a direct association. Pollutants, selective serotonin reuptake inhibitors, artificial insemination, and fertility medications may have a link, but results are often conflicted. Factors related to the delivery room experience may be associated with meconium aspiration syndrome, birth weight, and labor time. Several risk factors during the pregnancy and labor periods have been associated with autism; yet further studies with large populations are needed to establish definitive associations. The fact that several risk factors during the prenatal and labor periods are implicated in autism should prompt the medical community to focus on the pregnancy and labor periods as preventive measures to curb the incidence of autism.

## 1. Introduction

Autism is a complex condition that lacks an established method of prevention; thus there is a need for research to identify its possible causative factors. Identification of environmental factors is the first step in preventing and mitigating risks by primary prevention [1]. Statistical estimates show that one out of every 88 individuals in the United States is diagnosed with autism. The condition may be defined as a chronic neurodevelopmental disorder that first becomes prevalent in an individual during his/her early childhood. Consequently, autism results in a patient experiencing a life that is marked by the ongoing challenge of its negative effects on communication and interaction with others. The disorder is debilitating for the patient and immensely challenging for his/her family. An initial diagnosis of autism occurs as an individual is noticeably unable to achieve certain early childhood milestones in terms of communication and socialization. Furthermore, while the incidence and the prevalence of autism also tend to raise on an ever-increasing basis, public health concerns over the disorder have also been coupled with concerns over its etiology, which is still in the investigative stage of clinical research [2].

Initial research efforts, which were embarked upon during the 1950s, assert that complications in a given pregnancy, the birthing process, and the incidence of autism are linked. Over the past several decades, continuing research has been conducted, and much literature has been published that has supported the existence of the links between autism and factors that relate to pre-, peri-, and neonatal care. Such commonly identified, prenatal risk factors may include advanced-aged parents, a short gestation period, low birth weight, hyperbilirubinemia, and breech presentation [3]. Another key factor may be the use of any prenatal, prescription medication [4–6].

Autism, to reiterate, impairs an individual's ability to use language to effectively communicate and navigate social situations. The assertion that autism patients engage in repetitive, behavioral patterns has arguably become stereotyped. The symptoms of autism become notable in patient in the first three years of life and may necessitate lifelong care that must be provided by family members, guardians, or healthcare professionals. Autism and autism spectrum disorders (ASDs), including Asperger's syndrome and pervasive developmental disorder, have become increasingly diagnosed over the past decade. Accordingly, population studies, which have been conducted by the United States Centers for Disease Control and Prevention, have reported a prevalence of ASDs of 1 of 68 children at risk.

It should be noted, however, that, from a stand-alone perspective, each of the factors (as listed above) that link autism with pre-, peri-, and neonatal care does not correlate consistently as the incidence of autism is reviewed in terms of its cross-reference amongst various research studies. It should also be noted that, despite ongoing research efforts, relatively little has yet been divulged regarding delivery-room attributes that may be directly or indirectly associated with the incidence of autism. Furthermore, the nature of the precise catalyst of autism has not been revealed as of yet.

Early diagnosis of autism and any subsequent related intervention(s) may serve to alleviate the challenges that are faced by those individuals, who must manage the effects of autism on an ongoing basis, including patients, their guardians and/or families, and related healthcare professionals. The following narrative review aims to better understand the existing evidence for perinatal and labor-related associations with autism.

## 2. Discussion

The etiology of autism is unknown, although perinatal and neonatal exposures have been the focus of recent epidemiologic research [57]. Exposure to certain medications such as perinatal medications, antiepileptic drugs, supplements, and SSRIs has been studied as well as drug exposure to cocaine and smoking. Artificial insemination and fertility medications, meconium aspiration syndrome, and birth weight have also all been subjects of focus and the respective association with pregnancy and neurodevelopmental complications such as autism. Several studies have suggested the association between perinatal factors and the onset of autism. A recent case control study done in New South Wales, Australia, focused on determining risk factors for autism. Four factors were shown to be significantly associated with autism: being male, premature birth, maternal age greater than or equal to 35 years, and mothers born outside of Australia, with highest risk found in mothers from North-East or South-East Asia [7]. A separate study from Western Australia concluded that autistic children generally had parents who were older and had greater frequencies of threatened abortions, epidural anesthesia, labor induction, and labor duration of less than 1 hour. These children were more often born first, had a low Apgar score, and tended to have more complications [8]. Similar studies using a Swedish medical birth registry observed different factors that affected autism spectrum disorders. The researchers found that prematurity, low Apgar scores, growth restriction, or macrosomia, and if the mother was from sub-Saharan Africa or East Asia had a positive correlation with autism spectrum disorders [9]. Information obtained from the Finnish prenatal study of autism and autism spectrum disorders correlated advanced paternal age with an increased risk of ASD [10]. A group in Norway assessed the association between interpregnancy lengths and ASD and found that interpregnancy intervals for less than 9 months and interpregnancy between 9 and 11 months carried an increased risk for ASD [11].

Further evidence is suggested by other studies. Subjects used in an Australian study were diagnosed with autism spectrum disorder and compared to control subjects. The mothers of autistic children were studied, and it was demonstrated that the cases with autism had more pregnancy complications. The mothers had greater frequencies of threatened abortions, epidural caudal anesthesia use, labor induction, and labor duration of less than one hour. They were more likely to have experienced fetal distress, been delivered by an elective or emergency cesarean section, and had an Apgar score of less than 6 at one minute. It was concluded that although autism is unlikely to be caused by a single obstetric factor, there was an increased prevalence of obstetric complications among autism cases that is most likely due to underlying genetic factors and an interaction of these factors with the environment [8]. Another Australian study evaluated maternal conditions and perinatal characteristics associated with ASD and intellectual disability. Although a weak correlation was found between poor intrauterine environments and ASD with intellectual disabilities, there was no relationship found between ASD without intellectual disabilities and perinatal factors [12].

ASD may involve additional pathologies at the neuroanatomical level. A recent study illustrates that the neuropathology of some individuals who are diagnosed as having ASD may actually also have focal cortical dysplasias, which could explain the high incidence of sensory irregularities and seizures in ASD diagnosed patients [59]. Thus, focal cortical dysplasias may be a risk factor for ASD patients.

No specific article examined specific aspects such as room temperature, lack of equipment, time spent with the mother, or proximity to other neonates. However, some sources did cite other factors that could potentially be associated with the delivery room itself. These include the type of birth, such as whether it was breech, a prolonged birth, or an induced birth, along with various forms of fetal measures such as birth cry, fetal distress, low heart rate, or high fetal blood pressure [2, 3, 13–15, 60]. Further, a possible association exists with ventilation of neonates [16]. Feeding practices have also been looked at, as well as maternal states such as high temperature or an unhappy maternal emotional state [17–19, 58]. Lastly, the environmental impact of perinatal air pollutants has also been observed [20, 21]. Because these studies have pointed to all of these various factors which in and of themselves can all occur within a delivery room atmosphere, they warrant a further look into even more specific delivery room attributes to see if there is a more direct association between them and autism.

### 2.1. *β*2 Adrenergic Receptor Agonists

Beyond the cases conducted to study the relationship between pregnancy complications and augmented childbirth to autism, several studies have been done to investigate the association between prenatal exposures to *β*2 adrenergic receptor agonists to autism spectrum disorders. These agents include terbutaline, which is commonly used as a tocolytic and a bronchodilator in obstetrics. Terbutaline can cross the placenta and potentially affect the developing neonatal brain [22]. In a study of neonatal rats, Zerrate et al. found neuroinflammatory and behavioral changes, similar to that in autism, after administering terbutaline to subjects. The results demonstrated that terbutaline increased microglial activation in the subjects' cerebral cortex, cerebellar, and cerebrocortical white matter [23].

Nucleotide polymorphism of the *β*2 adrenergic receptor gene has also been implicated as risk factors for autism, namely, Gly 16 and Glu 27 [24, 25]. The researchers of independent subjects and dizygotic twins found that activation of receptors with either polymorphism has been link with increased signaling, secondary to decreased downregulation of the *β*2 adrenergic receptor gene. Critics of these genetic predisposition argued that since many genes are involved in the etiology of autism, the role of any single gene polymorphism in causing autism is small. The effect of each gene is further diluted by variable penetrance [61]. In clinical studies, researchers from Kaiser Permanente Northern California Hospitals examined 291 children born from 1995 to 1999, compared to 284 controls. They showed that there was no evidence linking *β*2 adrenergic receptor agonist exposure in pregnancy with an increase in autism risk, except exposure to terbutaline during the third trimester for more than two days [26, 62]. The authors suggested that future studies with larger sample sizes would be helpful to endorse or challenge their findings.

Based on aggregated evidence from animal and human studies, Witter et al. proposed that permanent changes in autonomic tone could result in neurological changes in the fetus, especially after two or more weeks of high dose *β*2 adrenergic receptor agonist exposure. These functional and behavioral changes may contribute to the signs and symptoms of autism [27].

### 2.2. Maternal Disease

In addition to environmental factors, infection and disease may play a role in the development of autism. Children born to mothers with antiphospholipid syndrome were observed to have a higher frequency of autism spectrum disorders in comparison to mothers without antiphospholipid syndrome, which further supports findings that perinatal complications increase the risk of autism in children [28]. Another study was organized to determine ASD in children born preterm and the role of exposure to perinatal inflammation. It was a review that covered the evidence supporting the idea that prenatal infection on the central nervous system may increase the incidence of autism spectrum disorders [63]. An increase in C-reactive protein indicates inflammation, which is common in infection. This increase in C-reactive protein has been found to have a correlation with a risk for autism spectrum disorders [29].

### 2.3. Antiepileptic Drugs

Although it has been shown that autism is unlikely to be caused by a single factor, there have been many studies conducted studying the relationship between the use of certain medications during pregnancy and the associated risk of autism. For example, the increased prevalence of the use of anticonvulsant medications during pregnancy is correlated with an increased risk of autism. Valproate is used for the treatment of epilepsy and other neuropsychological disorders and is considered one of the only available treatment options for women of childbearing potential [6]. Recent studies, however, have shown that exposure to valproate during pregnancy indicates a significantly increased risk of autism spectrum disorder, even after adjusting for maternal epilepsy. In fact, valproate use during pregnancy showed an 11.3% increased risk in developmental malformations, and it relied heavily on the dosage prescribed [64]. Women who were prescribed increased doses of valproic acid showed a twofold increase in risk of developmental malformations and significantly reduced intelligence [30]. Studies exposing pregnant rats and mice to valproate demonstrated increased autistic-like behaviors in the offspring. This behavior included social behavior deficits, increased repetitive behaviors, and deficits in communication. The animal studies performed by Roullet have been used as a model to ameliorate and improve upon studies that focus on the association between the risk of autism and the maternal need for anticonvulsants [5]. The current recommendation of health care professionals is to avoid anticonvulsant drug use such as valproate during pregnancy. It is important, however, to assess the intrinsic iatrogenic risk of birth defects or perinatal complications and the general safety for the expectant mother before discontinuing anticonvulsants [31].

### 2.4. Oral Supplements

Prenatal supplements are an important component of a healthy pregnancy. Prenatal folic acid supplements have been shown to reduce the risk of neural tube defects in children and are associated with a lower risk of autistic disorder [32]. In this study following 270 children, of the children whose mothers took folic acid, 0.1% developed autistic disorder [32]. Those children whose mothers were not exposed to folic acid comprised 0.21% of the group. This study also analyzed the use of fish oil supplements, which showed no association with autistic disorder [32]. Although no causality was found, it was determined that prenatal folic acid supplements taken during pregnancy were associated with a decreased risk of autistic disorder. Vitamin D, which has a unique role in brain homeostasis, embryogenesis and neurodevelopment, neural differentiation, and gene regulation, has recently been proposed as a possible environmental risk factor for autism during early childhood. Children with autism had significantly lower serum levels of 25-hydroxy vitamin D than healthy children. On the other hand, the involvement of maternal vitamin D during pregnancy in autism requires proper investigation. Recent studies have shown that a wide range of disorders, including neuropsychiatric disorders and autism, are associated with increased homocysteine levels in biological fluids. Homocysteine is an amino acid, which plays several important roles in human physiology. Various B vitamins such as B6 (pyridoxine), B12 (cobalamin), and B9 (folic acid) are required as cofactors by the enzymes involved in homocysteine metabolism. Therefore, it is crucial to monitor the homocysteine levels in body fluids of autistic children. It can provide information on genetic and physiological diseases, improper lifestyle (including dietary habits), and a variety of pathological conditions.

### 2.5. Illicit Drug Exposure, Smoking, and Pollutants

Drug exposure may have very serious complications during pregnancy. A study was conducted to evaluate the association between autism and developmental abnormalities in children with perinatal cocaine exposure. It was concluded that most of the cases had language delays and difficulties with expressive skills and communication disorders. Significant neurodevelopmental abnormalities were observed and a high frequency of autism was found in children exposed to cocaine, especially in comparison to those exposed to alcohol or opiates alone [33].

Smoking has been found to be an environmental hazard. Studies have been done determining the effects of smoking in a pregnant patient and the risk of autism in the mother's offspring. Epidemiological data were obtained from children with autism, including residential history of the mother, which was compared to the traffic-related air pollution assigned to each location using measures from the Environmental Protection Agency's Air Quality System data. It was assessed that maternal smoking was related to a modest increase in the risk of Pervasive Developmental Disorders, however, there was no known association between maternal smoking and childhood autism [34]. Interestingly, the amount of air pollution that a mother and child were exposed to including traffic related air pollution and nitrogen dioxide, both during pregnancy and during the first year of life, was associated with autism [35].

### 2.6. SSRIs

Selective serotonin reuptake inhibitors (SSRIs) are a class of drugs currently and increasingly prescribed for anxiety disorders and depression. These drugs, such as Fluoxetine, Sertraline, and Citalopram, work by inhibiting the reuptake of the neurotransmitter serotonin, resulting in a greater amount of serotonin at the synapse. Much like anticonvulsants and other drug exposures, the use of SSRIs in pregnant women and its association to autism have been explored. Previous studies have shown an increased blood level of serotonin in individuals affected with autism spectrum disorder, warranting the exploration of a possible association [65].

The risk of autism spectrum disorders was shown to be doubled with maternal use of SSRIs in a recent case controlled study of 298 children, with prenatal exposure reported for 20 of those children [36]. This was seen by evaluating medical records, considering both the SSRI exposure and the mental health history of the mother, and pointing to a high association with SSRI treatment and autism during the first trimester. However, the sample size for this study is small. A recent study conducted in Sweden by Rai et al. [37] expanded on this and illustrated a case control study of 4429 children with autism. It demonstrated an association between in utero exposure to both SSRIs and nonselective monoamine reuptake inhibitors with an increased risk of autism spectrum disorders, particularly without intellectual disability. However, there is again a limitation with interpreting medical records, such as prescribed versus actual use of SSRIs.

Further, other studies using animal models show that preclinical findings in rodents exposed to SSRIs during development point to an increase in depression and anxiety, exhibiting alteration in social behaviors in the offspring. The data show adverse effects of maternal mental illness on pregnancy outcomes and infant neurodevelopment but are not robust enough to discourage the use of SSRIs during human pregnancy [66]. In a study by Gur et al. [66], it was shown that the manipulation of serotonin during early development in both* in vitro* and* in vivo* models disturbs characteristic chemoarchitectural and electrophysiological brain features. This includes changes in raphe and callosal connections, sensory processing, myelin sheath formation, neophobia, and disrupted juvenile play behavior. These findings indicate that serotonin homeostasis is necessary for proper brain maturation and fetal development. Although SSRIs are not contraindicated for use during pregnancy, the effects should be examined more carefully [66]. In a similar study done by Simpson et al. [38], animal models were used to study the importance of serotonin homeostasis for the development of rat pups' brains. It was demonstrated that when the serotonin levels were altered by SSRIs during early development of brain structure, the functions were altered. This alteration was also more evident in males than in females. This suggests that SSRIs may have similar effects in human neonates [38].

Conversely, other recent studies have suggested that there may actually not be a strong association between SSRI intake and the subsequent development of autism. A recent cohort study of live births in Denmark from 1996 to 2005 involving 3,892 cases of ASD found no significant association, as only 52 cases of SSRI use were found [39]. Larger observational studies are required to establish a true link between the use of SSRIs and autism. Furthermore, it must be noted that the effect of depression itself as a factor in autism separate from the administration of SSRIs is a possibility. Thus, it may be that, in some cases, depression itself or other concomitant factors have a combined effect that is not necessarily due to the use of SSRIs.

### 2.7. Artificial Insemination and Fertility Medications

There is an increase in prevalence in the number of women utilizing fertility measures, thus calling for studies to determine the association between assisted reproductive therapy and neurodevelopmental complications, and more specifically autism. A nested case control study done by Lyall et al. [40] shows that assisted reproductive therapy and a history of infertility do not increase the risk for autism spectrum disorders. However, the age of the mother needs to be taken into consideration, as artificial insemination among older mothers has been studied to lead to more complications. Women under the age of 35 using fertility therapies such as ovulation inducing drugs and artificial insemination did not increase the risk for having a child with autism, while researchers suggest that the rise in fertility treatments in older women above 35 may increase the risk of having a child with autism for those using artificial insemination and/or ovulation inducing drugs. While the study is strong in that it uses a large national sample, it is important to note that both diagnoses and fertility treatments were self-reported [40]. Conversely, a recent cohort study conducted by Bay et al. showed a statistically significant increase in autism and other mental health disorders following ovulation induction [41]. This was done through a cohort study using 555,828 children in Denmark using information from a birth register and evaluated with statistical analyses. There were no significant associations found with* in vitro* fertilization/intracytoplasmic sperm injection (ICSI) or with cryopreserved embryos or gametes, types of hormones, or cause of infertility. The strength in this study includes long followup and low risk of selection bias, but numerous subgroup analyses may lead to risk of chance influencing significant associations.

In an epidemiological study conducted in India by Mamidala et al., maternal hormonal intervention was a significant risk factor for ASD, possibly due to maternal hormone disturbances causing obstetric complications, which in turn are a risk for ASD [42]. While there was a large sample size, there was an absence of detailed information on dosage and frequency of hormonal intervention.

Thus, with differing results it is evident that more specific studies need to be conducted on infertility treatments keeping in mind specific subtypes and their effect on autism in children. Controls for age can also be accounted for, and adverse effects can be more accurately studied in the future.

### 2.8. Meconium Aspiration Syndrome

Meconium aspiration syndrome, which occurs when a fetus who is under stress and not getting enough oxygen inhales waste products inside the womb, was linked to a sevenfold increase in the likelihood that a child would later develop autism. A study examined the incidence of autism in the neonatal intensive care unit and risk factors connected with autistic development. Findings in this study indicate that children with autism had a significantly higher history of meconium aspiration syndrome than the controls [43]. However, there is insufficient evidence to implicate any one perinatal or neonatal factor in autism etiology, although there is some evidence to suggest that exposure to a broad class of conditions reflecting general compromises to perinatal and neonatal health may increase the risk. Therefore meconium aspiration syndrome cannot be the cause of autistic disorder in children, but it can be a contributing factor [57].

### 2.9. Birth Weight

Many studies have evaluated the association between birth weight and the development of autism. A significant fourfold increased risk was observed in low birth weight females for autism accompanied by mental retardation. In low birth weight males there was no significant increased risk observed for autism alone. Though this implies a certain etiological premise of difference in gender, it is also important to note that while the population cohort design is strong, there was not a large number overall of the lower birth weight categories in females [44]. An earlier study by Wilkerson supports this finding as it determined that birth weight was a factor for the risk of autism spectrum disorders, with significant associations between autism and gestational age, maternal morphology and intrauterine stress. Specifically, it is the prescriptions taken during pregnancy, the length of labor, viral infection, abnormal presentation at delivery, and finally low birth weight that were associated with autism. In this study only 183 autistic children were considered and medical conditions of the mother based on a maternal self-report [18].

Further, several studies examined head circumference and height abnormalities in autism patients. Their studies suggest that the abnormal growth in children with autism spectrum disorders may be related to the causal factors that also increase the risk for ASD, as it was found that children with ASD had proportionally smaller head circumference compared to height during their first year of life, which was absent in premature/low birth weight children with ASD. Even more importantly, the body grew faster than the head for those with ASD by the end of the first year of life, suggesting a closer following of length to circumference during development. However, there may have been some recall bias and head circumference could only be measured on the first year of life all within a modest sample size, indicating a need for replicating the study in a larger cohort [45].

Similar studies were conducted illustrating that relative microcephaly was more frequent in children with ASD. However microcephaly can also be caused by other factors such as low intelligence and heredity, which could not be controlled for, and also only focused on measurements obtained at birth [46]. Expanding on this, a matched case control studies of children in Stockholm county showed a correlation between low growth for gestation age and preterm birth with ASD combined with intellectual disabilities. The strength of this study lies in its comprehensive sample and size but did not measure some other maternal factors such as substance abuse and nutritional influence [47].

Several other studies have focused on maternal changes during pregnancy and their association with autism. A population-based study in Utah (US) looked at prenatal variables of autistic children and focused on gestational weight. It was found that maternal prenatal weight gain, but not prepregnancy body mass index, is directly related to an increased risk of ASD. Because of use of cohort and control groups, it not only allowed a representative sample of Utah children, but also as a surveillance study could not directly assess cases and controls [48]. However, a recent longitudinal study using birth data from 4800 children from the United States showed that children born to over-/underweight mothers during prepregnancy had increased incidences of being born with a low birth weight. The group noted that low birth weight increased chances of developing ASD, as well as rapid head growth. This study concluded that abnormal maternal gestational weight indirectly caused an increased chance of developing ASD [49]. In addition to a lower birth weight population, a secondary analysis longitudinal design study using cranial ultrasound evidence of 14 study participants diagnosed with ASD identified that children with ventricular enlargement were strong indicators of the potential risk for autism spectrum disorders [50].

Overall, these birth weight studies illustrate that more research with larger populations needs to be conducted to determine whether autism etiology is genetic in relation to birth weight, whether birth weight is a risk factor or a cause of autism, how much mother's gestational age plays a role, and whether pre- or postpregnancy weight of the mother has a larger effect on prevalence of autism and ASD as research currently supports a variety of findings.

### 2.10. Labor Induction and Augmentation

Since the early 1990s, a number of studies have suggested the link between ASD and labor induction, such as the use of oxytocin [67]. The research that ensued found significantly different results between population-based studies and clinic based studies. Gardener et al.'s analysis showed induction increase in the risk of autism by 72% in three clinic based studies, while the selected population-based studies reported no association [57]. Recently, however, labor induction was positively correlated with autism in a large population study comparing birth records in North Carolina (1990–1998) and corresponding school records of autistic children (1997–2007). The study controlled for environmental factors such as socioeconomic status, maternal health, pregnancy-related events and conditions, and birth year. The authors reported that the link was particularly significant in males. The lack of differentiation of underlying complications in pregnancy, which lead to eventual use of induction, could be the cause of these conflicting findings [51]. Juul-Dam et al. summarized causes of prolonged labor, including “fetal malposition, fetopelvic disproportion, excess sedation, inadequate contraction, and rupture of fetal membranes before the onset of active labor.” However, these complications differ in severity and may require varied quantity of induction agent used at distinctive stages of labor. Further research is necessary to differentiate between the underlying obstetric complications that lead to the eventual use of induction and their respective potential association with autism [52].

### 2.11. Labor Time

A review of literature found limited number of researches on the topic of labor time and autism. The few identified studies abound with conflicting evidence. The research done by Glasson et al. purports that labor duration of less than 1 hour carries an increased risk of developing ASD [8]. On the other hand, Wilkerson et al. found that a prolonged labor (greater than 18–24 hours) resulted in an increased risk for autism [18]. One of the latest cohort studies on the topic that involved 268 subjects also suggests positive association between prolonged labor and autism [53]. However, among Gardener et al.'s meta-analysis of nine studies on this subject, five have neutral results, only three have positive association, and one has negative association [57].

Current understanding suggests that hypoxia is one of the complications of prolonged labor, along with infection, or head trauma from extended pressure. All of these complications could lead to permanent damages in the neonates' developing brain [52]. Neonatal oxygen deprivation can be quantified by several measurements, including low Apgar score, fetal distress, caesarian delivery, threatened abortion, and hemorrhage during pregnancy [68]. Four large cohort studies conducted across Sweden, Australia, and Denmark found that an Apgar score of 7 has a strong predictive value of autism [18, 54–56]; similar findings were reported by Garderner et al. [57]. Finally, Guinchat et al. [2] hypothesize that the increased prevalence of autism may be in part due to higher success in obstetric and neonatal care, which resulted in an increased survival rates of hypoxic neonates with brain damage.

In summary, some investigations have shown an association between length of labor and autism, but findings have been inconsistent owing to differences in sampling and methods. While there is still room for much further research, current understanding suggests that length of labor is possibly associated with autism. Prolonged delivery due to oversedation, inappropriate position of the fetus, and unbalanced fetopelvic size and oversedation, which results in induction, can play a role. It is also possible that children who become autistic may also be slow to deliver that the prolonged delivery is a symptom of, rather than a cause of, autism.

## 3. Conclusion

In summary, several attributes of the perinatal and labor experiences may be associated with the development of autism. Yet, importantly, these factors may work separately or may work together with other stimuli.  Table 1 provides a summary of the findings in the studies explored in this article.

While association between *β*2 adrenergic receptor agonists and autism spectrum disorders is not strong, exposure to terbutaline during the third trimester has shown to increase incidence. Furthermore terbutaline has been shown to increase microglial activation associated with behavioral abnormalities akin to autism. Labor induction and augmentation appear to be an area of association. While one study showed that children whose mothers received augmented and/or induced childbirth showed a greater risk for autism compared with controls, further research is needed to prove an association. The role of maternal disease cannot be ignored as antiphospholipid syndrome and perinatal inflammation may have important roles in the eventual development of autism. Antiepileptic drugs and oral supplements may play a role in later onset. Cocaine has been associated with an increase in the onset of neurodevelopmental abnormalities, but the direct influence on autism development is still lacking. While a link between smoking and autism could not be established, air pollution may have a role. Several studies have implicated SSRIs in autism, but a larger recent cohort study showed no association. Artificial insemination and fertility medications were largely found to have to link, yet reports are conflicted. Prolonged labor may also lead to neurological deficits and thus autism.

Attributes of the delivery room and birthing experience also may have a role in autism. While some evidence suggests that meconium aspiration syndrome may have a link as a contributing factor, more studies are needed. Birth weight, gestational age, and preterm birth may correlate with autism and intellectual disabilities.

The identification of prenatal factors that are linked to autism may serve as a paradigm shift in our current view and management of autism. The fact that several risk factors during the prenatal and labor periods are implicated in autism should prompt the medical community to focus on the pregnancy and labor periods as preventive measures to curb the incidence of autism.

## Figures and Tables

**Table 1:** Summary of autism studies.

Study	Summary/major findings
Bilder et al. [3]	Population study based on birth records in Utah (*n* = 138). Study found that an association between ASD and breech presentation may have a common etiology. Authors found that advanced maternal age and parity occurred more frequently in ASD children. Primary cesarean delivery was a significant perianal factor. No significant associations were established between neonatal factors and ASD.

Roberts et al. [4]	Population-based longitudinal cohort study with 116,430 women. Maternal abuse exposure in childhood was found to be directly correlated with increased risk of autism in offspring. Amount of abuse exposure also directly correlated with level of autism.

Christensen et al. [6]	Population study of all Denmark children born alive from the years 1996 to 2006. The study, which utilized national records, found a significant absolute risk between valproate exposure during pregnancy and autism in offspring.

Williams et al. [7]	Population-based data were used to examine risk factors for autism. Risk factors found to be significantly associated with autism were male gender, premature birth, maternal age greater than or equal to 35, mother not being born in Australia, and multiple birth.

Glasson et al. [8]	Association of autism spectrum disorders with perinatal obstetric factor was analyzed for a cohort of children. It was determined that a single obstetric factor is not likely to cause autism. Increased obstetric factors may be associated with autism because of underlying genetic and environmental factors.

Haglund and Källén [9]	Obstetrical information was reviewed for 250 children diagnosed with autism or Asperger syndrome using the Swedish Medical Birth Registry. Authors found that obstetric factors like prematurity, macrosomia, and low Apgar scores were associated with autism but not with Asperger syndrome. Authors also found positive association between autism and mothers born outside Nordic countries.

Lampi et al. [10]	Validity of the Finnish registry for diagnoses of autism was examined. A sample of 95 people from the Finnish Hospital Discharge Register who had been diagnosed with childhood autism or pervasive developmental disorder/pervasive developmental disorder not otherwise specified or Asperger's syndrome was examined. It was determined that the Finnish registry was valid for childhood autism diagnoses.

Gunnes et al. [11]	This study examined if there was an increased risk of autistic disorder in children born within a year of a sibling. Norway registry data were utilized to analyze 223,476 sibling pairs. An association between interpregnancy intervals less than one year and increased risk of autistic disorder was found.

Langridge et al. [12]	Study of 383,153 singletons born alive in Western Australia was conducted. Increased risk of autism spectrum disorder was found with the following factors: hypertension in pregnancy, asthma, antepartum hemorrhage (some types), breech presentation, need for resuscitation at birth, elective cesarean sections, preterm birth, and urinary tract infections.

Mamidala et al. [13]	A retrospective cohort of 942 children from India was examined. Twenty-five pre-, peri-, and neonatal risk factors of autism spectrum disorder were examined. Advanced maternal age, birth asphyxia, fetal distress, delayed birth cry, gestational respiratory infections, preterm birth, labor complications, and neonatal jaundice were found be significantly associated with increased risk of autism spectrum disorder.

Polo-Kantola et al. [14]	Seventy-one postmenopausal women were examined to evaluate estrogen replacement therapy and its effects on nocturnal periodic limb movements. It was found that estrogen replacement therapy does not alter nocturnal periodic limb movement incidence or intensity when it is given in doses used for climacteric symptoms.

May-Benson et al. [15]	Retrospective chart review was utilized to investigate 1000 children diagnosed with sensory processing disorder and 467 with autism spectrum disorder as well as sensory processing disorder. Jaundice was found to be three to four times more prevalent in sensory processing disorder and autism spectrum disorder children compared to typical children. Other factors found to be of higher incidence in these children were breech position, cord wrap and prolapse, assisted delivery methods, and high birth weight.

Kuzniewicz et al. [16]	A retrospective cohort of subjects born at greater than or equal to 24 weeks in Kaiser Permanente Northern California hospitals (*n* = 195,021) was investigated. Autism spectrum disorder was found to be about 3 times more prevalent in infants <27 weeks than in infants born term. Shorter gestation time was found to be directly associated with increased risk of autism spectrum disorder. Intracranial hemorrhage and high frequency ventilation were also associated with autism spectrum disorder for infants born <34 weeks.

Al-Farsi et al. [17]	A case control study was performed with 102 autism spectrum disorder subjects and 102 healthy control subjects. Association between late initiation of breastfeeding, bottle-feeding, prelacteal feeding, and nonintake of colostrum was found to be associated with autism spectrum disorder. Decreased risk of autism spectrum disorder was found with exclusive breastfeeding and continued breastfeeding.

Wilkerson et al. [18]	The authors conducted a study involving mothers of 183 mothers of autistic children and 209 mothers of normal children. The mothers in the study completed a Maternal Perinatal Scale which surveyed complications of pregnancies and medical conditions. It was found that 65% of autistic cases could be grouped using differences in gestational age, maternal morphology, and intrauterine stress. In addition, urinary tract infection, high temperatures, and depression also contributed to the separation of groups.

Zhang et al. [19]	A case control study was conducted of 190 children in China with and without autism. Nine risk factors were found to be associated with autism: maternal second hand smoke exposure, advanced paternal age at delivery (>30 years of age), maternal medical conditions not related to the pregnancy, gravidity >1, mother being unhappy, nuchal cord, gestational complications, and edema.

Volk et al. [20]	A study conducted with 252 cases of autism spectrum disorder and 156 control subjects was examined. MET rs1858830 CC genotype as well as air pollution exposure may increase risk of autism spectrum disorder.

Roberts et al. [21]	A study was conducted that examined the association of air pollution exposure and autism spectrum disorder. This has 325 cases and 22,101 controls. Significant association between air pollutants and autism spectrum disorder was found.

Bergman et al. [22]	This study examined 22 women who were given terbutaline intravenously before delivery and were delivered by elective cesarean section. The authors found that venous umbilical blood slowly but continuously uptook terbutaline.

Zerrate et al. [23]	This study examined the effects of terbutaline on newborn rats to determine if terbutaline is a risk factor for autism spectrum disorder. The authors found that overstimulation of the beta-2 adrenergic receptor, which terbutaline is an agonist for, is associated with behavioral abnormalities which are similar to that of autism.

Connors et al. [24]	Dizygotic twin data were analyzed for association of autism spectrum disorders and terbutaline exposure. It was found that continuous exposure for 2 weeks or more of terbutaline has an increased risk for autism spectrum disorders in twins that are dizygotic. In addition, a significant association was found between 16G and 27E polymorphisms in the beta-2 adrenergic receptor and autism.

Cheslack-Postava et al. [25]	It was examined if there was association with the ADRB2 allele for the gene coding for the beta-2 adrenergic receptor and autism. This study found that the Glu27 allele of the ADRB2 gene may be involved with increased risk of autism.

Croen et al. [26]	A case control study with children born from the years 1995–1999 at hospitals in California was conducted to examine the association between autism spectrum disorder and prenatal exposure to beta-2 adrenergic receptor agonists like terbutaline. It was found that terbutaline was the only beta-2 adrenergic receptor agonist that was associated with increased risk of autism spectrum disorder, but only when exposure was greater than or equal to 2 days during the third trimester.

Witter et al. [27]	The authors of this paper found that overstimulation of the beta-2 adrenergic receptor during certain periods of time in prenatal development may have an association with autism spectrum disorder due to induced behavioral and functional teratogenesis.

Abisror et al. [28]	This retrospective study compared outcomes of babies born from mothers diagnosed with primary antiphospholipid syndrome (*n* = 26) and mothers diagnosed with systemic lupus erythematosus (*n* = 9). Autism spectrum disorder was found in the offspring of mothers diagnosed with antiphospholipid syndrome.

Brown et al. [29]	A cohort study was conducted that examined inflammation during pregnancy and correlation with autism spectrum disorder of offspring. Significant association was found between offspring autism and increased C-reactive protein levels in the mother.

Banach et al. [30]	This study investigated the relationship between autism and gender as well as nonverbal IQ. Single child daughters with autism showed lower nonverbal IQ than single child sons with autism. There appears to be variability in nonverbal IQ and gender in autism.

Rasalam et al. [31]	The authors of this study explored in long term in utero exposure to anticonvulsant drugs and risk of autism using 260 children. An association between in utero exposure to anticonvulsant drugs and autism spectrum disorder development was found.

Surén et al. [32]	A population-based study with 85,176 children was conducted to examine relationship between prenatal supplements of folic acid and risk of development of autism spectrum disorder. The authors found an association between folic acid supplementation during pregnancy and decreased risk of autism spectrum disorder in offspring.

Davis et al. [33]	A study utilizing records of 70 subjects exposed to cocaine in utero was conducted to examine developmental abnormalities. Autism was found in high frequency in offspring of mothers using cocaine during pregnancy (11.4%).

Tran et al. [34]	This was a population-based study that utilized the Finnish Medical Birth Register. The study examined risk of autism spectrum disorder in offspring of mothers who smoke tobacco during the entire length of pregnancy. The authors found no association between autism and maternal smoking.

Volk et al. [35]	The authors of this paper conducted a population-based study that was case controlled and looked for relationships between air pollution created by traffic, quality of air, and autism. The study had 279 subjects with autism and 245 control subjects. An association was found between autism and exposure to pollution caused by traffic.

Croen et al. [36]	This population-based case controlled study attempted to determine association between maternal antidepressant utilization during pregnancy and autism spectrum disorder in offspring. In utero exposure to antidepressants may be associated with autism development, but the authors suggest that more studies should be conducted.

Rai et al. [37]	The authors conducted a population-based case controlled study and examined risk of autism spectrum disorders in offspring of mothers using serotonin selective reuptake inhibitors and tricyclic antidepressants in pregnancy. An increased risk of autism was found in children exposed in utero to serotonin selective reuptake inhibitors and tricyclic antidepressants.

Simpson et al. [38]	This study utilized rats and examined serotonin levels and how alterations in serotonin may affect neurodevelopment and development of autism. Cortical organization of rats was examined after serotonin selective reuptake inhibitor exposure pre- and postnatally. Disruptions in brain features were found as well as changes in behavior. The authors call for further research of serotonin selective reuptake inhibitor exposure and autism development in humans.

Hviid et al. [39]	A study utilizing 3892 subjects diagnosed with autism spectrum disorder was examined for association between serotonin selective reuptake inhibitor exposure in utero and autism spectrum disorder development. Significant association was not found but further studies are warranted.

Lyall et al. [40]	This was a nested case control study that utilized the Nurses' Health Study II. The analysis found that therapy for reproductive assistance as well as infertility history did not show an increased risk of autism spectrum disorder in offspring unless the mother was 35 years of age or older.

Bay et al. [41]	This was a cohort study that used the Danish National Health Registers to investigate association between fertility treatments and autism spectrum disorder, as well as other mental disorders. A significant increase in risk of autism spectrum disorder was seen in offspring of mothers who utilized fertility treatments, but the increase in risk was low.

Mamidala et al. [42]	A study was conducted in India to explore hormonal interventions and the association with autism spectrum disorder. The study utilized 942 children, 471 of whom had been diagnosed with autism spectrum disorders. Analysis of data collected revealed that hormonal intervention used by the mother significantly increased the risk of offspring having autism spectrum disorder.

Matsuishi et al. [43]	The authors investigated the prevalence of autistic disorder diagnosed in children at St. Mary's Hospital. The number of children identified having autistic disorder was higher than previously reported values in the nation of Japan.

Schendel and Bhasin [44]	A retrospective cohort study was conducted that compared weight at birth and gestational age of born children to rates of autism. Autism was found less than other developmental disabilities in preterm children and in low birth weight term children. However, autism did appear more when the child was also intellectually disabled.

Schrieken et al. [45]	This study used 96 children with autism spectrum disorder and a control of 163 children to examine if abnormalities in growth may be linked to autism spectrum disorder. Autism spectrum disorder was seen more in children who were first born or premature or had low birth weight. Results suggested that autism spectrum disorder may have a different reason for abnormal growth than premature/low birth weight children without autism spectrum disorder due to a difference in head circumference compared to height in the two groups of children.

Grandgeorge et al. [46]	The study utilized 422 children with autism spectrum disorder and 153 control children and measured attributes of these children. A significant finding of increased incidence of macrocephaly and microcephaly in autism spectrum disorder children was found.

Abel et al. [47]	A Scandinavian population study was performed that examined the relationship between growth of the fetus and autism spectrum disorder development. It was found that difference in fetal growth either too high or too low may be significant for development of autism spectrum disorder.

Bilder et al. [48]	This was a population-based autism spectrum disorder cohort study (*n* = 128). Weight gain during pregnancy was found to be significantly associated with increased risk of autism spectrum disorder in offspring. However, prepregnancy weight and body mass index was not significantly associated with increased risk of autism spectrum disorder in offspring.

Moss and Chugani [49]	This study investigated prepregnancy weight and its relationship to autism in offspring. The authors found increased incidence of autism in children with very low birth weight, which correlated with mothers that were underweight or overweight.

Movsas et al. [50]	A secondary analysis of a study was performed to explore the link between abnormalities seen in the craniums of neonates via ultrasound and subsequent risk of autism spectrum disorder. It was found that particular types of white matter injury were associated with increased risk of autism spectrum disorder diagnoses, as well as ventricular enlargement.

Gregory et al. [51]	This study investigated the relationship between risk of autism diagnosis in induced and/or augmented births and births that were not. A total of 625,042 subjects were studied, of which greater than 5500 were documented as being autistic. The authors found increased incidence of autism diagnosis in induced births as well as augmented births.

Juul-Dam et al. [52]	This study investigated pre-, peri-, and neonatal factors in children that were diagnosed as autistic and compared these factors' incidence with a control population. Most factors had about the same incidence between the two populations. However, increased incidence of uterine bleeding and decreased incidence of contraceptive use as well as decreased incidence of infection of the vagina of the mother were associated with autistic children.

Maramara et al. [53]	A study was conducted with 268 subjects in New Jersey diagnosed with autism spectrum disorder. A questionnaire was given to the subjects and some factors were compared to the New Jersey population statistics. It was found that the subjects of the study had significantly higher incidence of the following factors: advanced maternal age of 35 years or older, decreased weight at birth, hypoxia, long labor time, bleeding from the vagina, multiple gestation, and prematurity.

Eaton et al. [54]	Records from Denmark were used for this study, which attempted to find the relationship between obstetric complications and psychopathology risk with a focus on autism. The authors found an association and determined that birth weight and growth rate as well as Apgar score taken at 5 minutes were the best predictors of increased risk.

Hultman et al. [55]	This nested case control study utilized the Swedish Birth Register to utilize information of 408 children diagnosed with autism and examined perinatal risk factors. Associations were found between autism diagnosis and smoking during pregnancy, birth of the mother outside of the regions of North America and Europe, delivery via cesarean section, a low Apgar score taken at 5 minutes, congenital malformations, and being small for gestational age. Associations were not found between autism and circumference of the head, being one of twins, diabetes of the mother, or season during which birth took place.

Larsson et al. [56]	This case control study looked at the relationship between autism and perinatal factors, socioeconomic status, and the psychiatric history of parents. Data were used from the Denmark registries. No significant relationship was found between autism and socioeconomic status, weight for age of gestation, age of the parents, parity, and visits before birth. However, association of autism and environmental factors prenatally and the psychiatric history of parents was found.

Gardener et al. [57]	A meta-analysis was conducted using research available on three databases (PsycInfo, PubMed, and Embase) and 40 studies were utilized. More than 60 neonatal and perinatal risk factors were examined for their relationship to autism, and many were found to be associated. These included nonnormal presentation, hyperbilirubinemia, complications with the umbilical cord, Rh antigen or blood type incompatibility, distress with the fetus, anemia of the neonate, injury or trauma during birth, aspiration of meconium, multiple birth, difficulties with feeding, hemorrhage of the mother, nonoptimal 5-minute Apgar score, and congenital malformations.

Russell et al. [58]	This was a retrospective study that analyzed a UK cohort to investigate if social factors and demographic factors may be part of the diagnosis of autism spectrum disorder in children who are diagnosed. It was found that boys were more likely to be diagnosed with autism spectrum disorder than girls with similar symptoms.

Casanova et al. [59]	The purpose of this study was to examine brains of subjects previously diagnosed with autism spectrum disorder for abnormal thinning of the cerebral cortex as well as neuronal morphometric abnormalities. Foci of decreased width of cortex were found in brains of autism spectrum disorder individuals, as well as a decrease in size of the neurons of affected locations. The pathology seen correlates with focal cortical dysplasia diagnosis, which may provide an explanation for the high incidence of seizures in individuals with autism spectrum disorder.

## Abbreviations

ASD: Autism spectrum disorder
SSRI: Selective serotonin reuptake inhibitor
